# Surgical treatment of Ebstein anomaly: combined correction of morphologic alterations and arrhythmias

**DOI:** 10.1186/1749-8090-8-S1-O324

**Published:** 2013-09-11

**Authors:** Leonardo A Mulinari

**Affiliations:** 1 Head of Division of Cardiothoracic Surgery, Federal University of Parana, Curitiba, Parana, Brazil

## 

Ebstein’s anomaly corresponds to less than 1% of all cardiac congenital malformations. In this anomaly, there is a fail in delamination of the posterior and septal cusps of the tricuspid valve during embryogenesis, resulting in adherence of these cusps to the subjacent myocardium, rotation and apical displacement of the functional valvar annulus. This anatomical changes lead to valve dysfunction, atrialization of part of the right ventricle and dilatation of the right atrioventricular junction, with consequent impairment in the ventricular function. The apical displacement of the cusps leads to discontinuity between the fibrous valvar skeleton and the functional valvar annulus, creating an anatomic substrate for accessory conduction pathways and ventricular pre-excitation. This is reflected in the high prevalence of pre-excitation and Wolf-Parkinson-White (WPW) syndrome in these patients, with consequent increase in risk of supraventricular and ventricular tachiarrhytmias, syncope and sudden cardiac death.

Elimination of the arrytmogenic substrate by means of surgery or catheter results in significant reduction in mortality and morbidity in the medium and long-term. Nevertheless, ablation, especially by means of catheter, still faces important challenges when performed both prior and subsequent to surgical correction. Before the surgical repair, the ablation is dificulted by the anatomic particularities of this malformation, such as right ventricular dilatation changing the usual landmarks, presence of tricuspid regurgitation, greater catheter instability and often presence of multiple and broad accessory pathways. After the surgical correction, there are also marked technical challenges involving catheter ablation due to the inaccessibility of the accessory pathway to the intravenous catheter, as it becomes “buried” by the surgical approach involving the tricuspid ring. These factors together reduce the success rate of catheter ablation in patients with Ebstein’s anomaly compared with other patient groups without this malformation. In an attempt to reduce risks and increase success rate in eliminating the arrytmogenic substrate, some groups have performed a combined procedure in patients with surgical indication, where the electrophysiological study and eventual ablation are performed during the surgical correction.

The aim of the present study is to report our experience with the combined treatment, with emphasis on the prevalence of arrhythmias during the in-hospital period in this group of patients.

## Methods

We performed a retrospective observational data collection from all patients who underwent surgical correction of Ebstein’s anomaly with a biventricular or “one and a half” ventricle approach between April 2005 and March 2013 at Pequeno Principe Children’s Hospital by the same surgical team.

The collected data were: related to the surgical procedure, such as aortic cross clamp time and extracorporeal circulation time; related to the immediate postoperative period, such as mechanical ventilation time, use of vasoactive substances, length of stay in intensive care; related to the prevalence of arrhythmias during hospital stay, including need for definitive cardiac stimulation (pacemaker with or without cardioverter defibrillator); related to the surgical mortality (first 30 days after surgery).

Intra-operative electrophysiological study, when performed, took place subsequently to sternotomy. Catheters were positioned in the tricuspid annulus, right atrium and left ventricle for stimulation and collection of epimyocardial electrocardiograms (ECG’s). Continuous atrial stimulation was performed until the atrioventricular (Wenckebach) point was achieved. Subsequently, the slow ventricular stimulation protocol was applied for investigation of abnormal retrograde conduction pathways.

When anomalous pathways were identified, an ablation catheter was positioned in the pathway’s location, and radiofrequency was applied in this region in one lesion line with 20-60 W of power, temperature between 40-50° C, impedance around 120-180 Ω and duration between 30 and 70 seconds. Subsequently, a new ventricular stimulation test was performed, where the disappearance of conduction through the accessory pathway was often observed.

The surgical technique applied for correction of the anatomical abnormalities was naturally individualized according to each patient’s anatomical characteristics. Overall, extracorporeal circulation was established with moderate hypothermia at 32°C. The aorta was cross clamped and cardioplegy solution at 4°C was infused. Right atriotomy was performed. Tricuspid valvuloplasty using the cone or Carpentier techniques was performed. In case the valvar characteristics did not favour valve repair, valve replacement using biological prosthesis was undertaken. Closure of the atrial septal defect was performed using a patch of pericardium (autologous or bovine) or Goretex®. In patients with hypoplastic right ventricle, right cavopulmonary anastomosis (Glenn procedure) was performed according to the usual technique.

This study was approved by the local ethical committee and performed according to recommendations stated in Helsinki II declaration.

We will present the results and conclusion of this study.

